# A glucose-insulin-glucagon coupled model of the isoglycemic intravenous glucose infusion experiment

**DOI:** 10.3389/fphys.2022.911616

**Published:** 2022-09-06

**Authors:** Vijaya Subramanian, Jonatan I. Bagger, Jens J. Holst, Filip K. Knop, Tina Vilsbøll

**Affiliations:** ^1^ Institute for Computational Medicine, Johns Hopkins University, Baltimore, MD, United States; ^2^ Center for Clinical Metabolic Research, Herlev and Gentofte Hospital, University of Copenhagen, Hellerup, Denmark; ^3^ Novo Nordisk Foundation Center for Basic Metabolic Research, Faculty of Health and Medical Sciences, University of Copenhagen, Copenhagen, Denmark; ^4^ Clinical Research, Steno Diabetes Center Copenhagen, Herlev, Denmark; ^5^ Department of Biomedical Sciences, Faculty of Health and Medical Sciences, University of Copenhagen, Copenhagen, Denmark; ^6^ Department of Clinical Medicine, Faculty of Health and Medical Sciences, University of Copenhagen, Copenhagen, Denmark

**Keywords:** glucagon action, glucagon suppression, glucagon secretion, insulin sensitivity, insulin secretion, hysteresis, type 2 diabetes

## Abstract

Type 2 diabetes (T2D) is a pathophysiology that is characterized by insulin resistance, beta- and alpha-cell dysfunction. Mathematical models of various glucose challenge experiments have been developed to quantify the contribution of insulin and beta-cell dysfunction to the pathophysiology of T2D. There is a need for effective extended models that also capture the impact of alpha-cell dysregulation on T2D. In this paper a delay differential equation-based model is developed to describe the coupled glucose-insulin-glucagon dynamics in the isoglycemic intravenous glucose infusion (IIGI) experiment. As the glucose profile in IIGI is tailored to match that of a corresponding oral glucose tolerance test (OGTT), it provides a perfect method for studying hormone responses that are in the normal physiological domain and without the confounding effect of incretins and other gut mediated factors. The model was fit to IIGI data from individuals with and without T2D. Parameters related to glucagon action, suppression, and secretion as well as measures of insulin sensitivity, and glucose stimulated response were determined simultaneously. Significant impairment in glucose dependent glucagon suppression was observed in patients with T2D (duration of T2D: 8 (6–36) months) relative to weight matched control subjects (CS) without diabetes (k_1_ (mM)^−1^: 0.16 ± 0.015 (T2D, *n* = 7); 0.26 ± 0.047 (CS, *n* = 7)). Insulin action was significantly lower in patients with T2D (a_1_ (10 pM min)^−1^: 0.000084 ± 0.0000075 (T2D); 0.00052 ± 0.00015 (CS)) and the Hill coefficient in the equation for glucose dependent insulin response was found to be significantly different in T2D patients relative to CS (h: 1.4 ± 0.15; 1.9 ± 0.14). Trends in parameters with respect to fasting plasma glucose, HbA1c and 2-h glucose values are also presented. Significantly, a negative linear relationship is observed between the glucagon suppression parameter, k_1_, and the three markers for diabetes and is thus indicative of the role of glucagon in exacerbating the pathophysiology of diabetes (Spearman Rank Correlation: (*n* = 12; (−0.79, 0.002), (−0.73,.007), (−0.86,.0003)) respectively).

## 1 Introduction

Glucose homeostasis is maintained primarily by the action of the two pancreatic hormones, insulin and glucagon, in conjunction with a host of other modulators. (Röder et al., 2016). Beta- and alpha-cell dysfunction both contribute to the pathophysiology of type 2 diabetes (T2D). (Burcelin et al., 2008; Ashcroft and Rorsman, 2012; Cryer, 2012; Cerf, 2013; Godoy-Matos, 2014; Moon and Won, 2015; Eizirik et al., 2020). Reduced insulin secretion from the pancreatic beta-cells and reduced insulin sensitivity in various tissues in the body lead to high postprandial glucose excursions. (DeFronzo and Tripathy, 2009; Montanya, 2014; Titchenell et al., 2016; Santoleri and Titchenell, 2019). In addition, higher basal levels of glucagon and impaired suppression of glucagon secretion is implicated in elevated fasting and post-prandial glucose levels in individuals with T2D. (Unger and Orci, 1975; Gerich, 1988; Dunning and Gerich, 2007; Lee et al., 2011). Theoretical models of glucose, insulin and glucagon dynamics can be used to quantify the extent of dysregulation in hormonal control of glucose homeostasis in T2D by fitting the models to data from various glucose challenge experiments. (Bergman et al., 1979; Bergman et al., 1981; Mari et al., 2002a; Dalla Man et al., 2002; Ferrannini et al., 2005; Dalla Man et al., 2006; Panunzi et al., 2007; Palumbo et al., 2013; Kelly et al., 2019; Bergman, 2021; Morettini et al., 2021).

The oral glucose tolerance test (OGTT) and the intravenous glucose tolerance test (IVGTT) have been used to quantify different aspects of plasma glucose regulation. (Panunzi et al., 2007; Cobelli et al., 2014; Bergman, 2021). The advantage of the OGTT is that it represents a physiological response to oral ingestion of nutrients. The challenge from a mathematical modeling point of view is that stimulation of the gut results not only in glucose dependent insulin secretion but also numerous confounding factors, e.g., the incretin effect. (Nauck et al., 1986; Knop et al., 2007; Nauck and Meier, 2016). Gut mediated effects do not come into play when glucose is administered intravenously. In a typical IVGTT, both first phase and second phase insulin secretion are observed in response to glucose challenge. (Bergman et al., 1981; Caumo and Luzi, 2004). Another method for studying glucose-insulin-glucagon dynamics is the isoglycemic intravenous glucose infusion (IIGI), which matches the glucose excursion observed during an OGTT but does not stimulate incretin secretion. (Bagger et al., 2011; Bagger et al., 2014; Nauck and Meier, 2016). Historically, IIGI has been used to obtain a quantitative measure of the incretin effect based on the differential insulin response observed in the OGTT and the corresponding IIGI experiment. In an IIGI, the typical first phase insulin response followed by the slower second phase of the bolus IVGTT is not observed. Instead, a single phase that tracks glucose concentration is observed. The shape of the insulin response is closer to that observed during oral ingestion because the delivery of glucose to the beta cells mimics normal physiological graded delivery from oral glucose administration. (Caumo and Luzi, 2004). Thus, the data from such experiments can be used to estimate parameters of glucose dependent insulin response in addition to insulin sensitivity by fitting a suitable minimal model of glucose regulation without confounding factors from the gut.

While the role of insulin mediated regulation of glucose homeostasis is well established and the contribution to the pathophysiology of T2D has been extensively quantified (Mari et al., 2002b; Panunzi et al., 2007; Cobelli et al., 2009; Cobelli et al., 2014; Bergman, 2021), the role of glucagon and alpha-cell dysregulation is less well studied from a computational perspective. Models have been developed to study glucagon secretion from the alpha cells or pancreatic islets addressing glucose dependent intrinsic and paracrine regulation. (Diderichsen and Göpel, 2006; Fridlyand and Philipson, 2012; Watts and Sherman, 2014; Briant et al., 2016; Watts et al., 2016; Briant et al., 2018; Zmazek et al., 2021). At the whole-body systems level, glucagon dynamics has been included in complex models that describe regulation of glucose homeostasis by the interplay between different organ systems. (Cobelli et al., 1982; Sulston et al., 2006; Kim et al., 2007; De Gaetano and Hardy, 2019). These models included many coupled differential equations and large number of parameters which make them less amenable to validation based on data from glucose challenge experiments for example. On the other hand, minimal models such as those developed for assessing insulin sensitivity and beta cell function are particularly useful in highlighting the contribution of specific impairments to the pathophysiology of diabetes and are more easily validated with data. (Bergman et al., 1979; Mari et al., 2002a; Dalla Man et al., 2002; Ferrannini et al., 2005; Panunzi et al., 2007; Bergman, 2021). The drawback with the glucose-insulin models is that they do not include the dynamics of the counter-regulator glucagon in establishing glucose homeostasis. A more complete minimal model which includes glucagon dynamics coupled to insulin and glucose dynamics would be self-consistent and yield information on glucagon action, secretion and suppression in addition to insulin related parameters. A few minimal models have included glucagon dynamics during IVGTT and OGTT respectively. (Kelly et al., 2019; Morettini et al., 2021). Glucagon dynamics has been described differently in each of the previous models (complex and minimal) particularly with respect to the regulation of glucagon secretion and suppression. In the paper by Morettini et al. (2021), a glucagon-c-peptide coupled model which did not include glucose dynamics was developed to describe suppression of glucagon secretion during OGTT. As the model did not include glucose dynamics, parameters related to glucagon action and secretion, insulin sensitivity, and secretion could not be determined simultaneously. In the IVGTT minimal model, (Kelly et al., 2019), the dynamics of glucose, insulin and glucagon were all included. In the description of glucagon dynamics, glucagon suppression is assumed to be linearly dependent on plasma insulin concentration and glucagon secretion occurs only when glucose levels drop below baseline. Experimental evidence from human islet level studies indicates that glucagon suppression at low glucose is controlled primarily through intrinsic regulation by glucose. (Tian et al., 2011; Walker et al., 2011; Yu et al., 2019). At high glucose, the intrinsic regulation is modulated by glucose dependent paracrine effects mediated by somatostatin. (Briant et al., 2016; Briant et al., 2018). In the paper by Elliot et al., (Elliott et al., 2015), insulin and somatostatin have been shown to act synergistically in regulating glucagon concentrations at high glucose in human islets. In the hypoglycemic range Bolli et al. (1984) have shown that glucagon secretion is regulated exclusively by glucose. Though the nature of paracrine regulation and the factors that mediate it are uncertain there is consensus on the observation that it occurs in a glucose dependent manner. In the OGTT model, (Morettini et al., 2021), glucagon suppression is attributed exclusively to insulin, ignoring intrinsic regulation by glucose. In the IVGTT minimal model, (Kelly et al., 2019), glucagon suppression is again attributed to insulin at glucose levels above baseline. In the comprehensive models, insulin dependent hyperbolic tangent functions, (Cobelli et al., 1982), quadratic functions, (Kim et al., 2007), and inverse functions (Sulston et al., 2006) have been used to describe glucagon suppression but it is unclear why the particular forms were chosen.

In this paper, a parsimonious model based on delay differential equations, that extends previous insulin-glucose models (Panunzi et al., 2007) was developed to include glucagon dynamics. The coupled model allows for the determination of parameters related to both insulin and glucagon regulation of glucose homeostasis in one step. Glucagon and insulin response to glucose are modeled on dose response data from human islet level studies of alpha and beta cell secretion in contrast to previous models. (Walker et al., 2011). The glucagon dynamics is described by a phenomenological model based on the data from IIGI experiments. Glucagon secretion and suppression are shown to be regulated by glucose as in reference (Walker et al., 2011; De Gaetano and Hardy, 2019) but the magnitude of the suppression is varied during the course of the dynamics. This allows for the description of the prolonged suppression of glucagon secretion and resulting delayed recovery to baseline as observed in the data which is likely due to paracrine effects. The model thus incorporates intrinsic and possible paracrine regulation in a glucose dependent manner and is described in detail in the methods section.

The model developed is fit simultaneously to glucose, insulin and glucagon data from IIGI experiments on individuals with T2D and without diabetes (CS) previously published in the papers by Bagger et al. among others. (Bagger et al., 2011; Mari et al., 2013; Bagger et al., 2014; Alskär et al., 2016; Guiastrennec et al., 2016; Røge et al., 2017; Tura et al., 2017). There are significant advantages of fitting IIGI over OGTT data namely: 1) there are fewer parameters in the model as exogenous glucose arrival is a known quantity unlike in an OGTT; 2) hormone secretory and suppression parameters determined are free of gut mediated effects; 3) parameters that could not be estimated from fitting OGTT data, because of gut stimulation can be determined from IIGI, such as the Hill coefficient in the glucose dependent insulin response; 4) the data from the IIGI experiments also reveal unusual behavior in the insulin response in T2D patients such as significant time delays in insulin secretion, quantification of which would give another tool to distinguish between T2D and control subjects (CS); and 5) there have also been questions regarding insulin response contributing to post prandial glucose lowering below baseline, a phenomenon observed particularly when exogenous glucose loads are high. (Saha, 2006; Parekh et al., 2014). A related pathophysiology is reactive hypoglycemia where glucose levels drop well below baseline and patients present with the Whipple’s triad. (Ahmadpour and Kabadi, 1997; Brun et al., 2000; Suzuki et al., 2016). If there is a lag in insulin return to baseline, i.e., if high levels of insulin secretion persist after plasma glucose levels start dropping, then it would explain postprandial glucose lowering. Modeling the glucose dependent insulin response using a hysteresis model should reveal if a lag in insulin recovery to baseline levels exists and causes postprandial hypoglycemia.

In this paper, the role of alpha- and beta-cell dysfunction in T2D is quantified and highlighted. The question of whether hysteresis in insulin secretion plays a role in postprandial hypoglycemia is also addressed. In addition, correlations between the parameters determined and the hallmarks of T2D, fasting plasma glucose (FPG), hemoglobin A1c (HbA1c) and 2-h plasma glucose (2 h PG) values are presented and highlighted.

## 2 Modeling and data analysis

### 2.1 Glucose-insulin-glucagon model

In this paper, a parsimonious model that includes glucagon dynamics was developed to describe the coupled glucose-insulin-glucagon system and is presented in Eqs 1–3. The model is an extension of the delay differential equation model of Panunzi et al. (Panunzi et al., 2007; De Gaetano et al., 2008). Equation 1 describes glucose dynamics. The rate of change of glucose is given by a source term depending on glucagon and the exogenous glucose infused during the IIGI experiment and clearance terms depending on glucose and insulin. The first term in Eq. 1 represents glucose dependent glucose clearance as in the Bergman model (Bergman et al., 1979) and is first order in glucose with rate constant S_G_. The second term represents insulin dependent glucose clearance and is first order in insulin and glucose. The rate constant a_1_ gives a measure of insulin sensitivity; it is analogous to the parameter S_I_ in the Bergman minimal model and K_xgI_ in the paper by Panunzi et al. Hepatic glucose production is assumed to be driven primarily by glucagon and is given by the third term in Eq. 1. It is first order in glucagon concentration and the rate constant a_2_ gives a measure of glucagon action in the liver. Hepatic glucose production would likely also depend on other substrates such as glycogen in glycogenolysis, but they are assumed to be in excess and the pseudo first order (Keeler et al., 2018) dependence on glucagon used should be sufficient. In the model of De Gaetano et al., (De Gaetano and Hardy, 2019), glucagon is included in the fast dynamics, but they use saturation kinetics to describe glucagon-dependent hepatic glucose production while a first order dependence is used in the paper by Kelly et al. (2019) As the extent of insulin dependent suppression of hepatic glucose production is uncertain, it was not included in this model (Gastaldelli et al., 2001; Adkins et al., 2003; Kaplan et al., 2008). The rate of glucose arrival in the plasma, R_IIGI,_ is determined from the glucose infusion rate, G_infusion_, during the IIGI as shown in Eq. 6. In the underlying experiments, the glucose infusion was manually adjusted in the IIGI protocol to match the OGTT profile. The average amount of glucose infused every 15 min was used to approximate the actual glucose infusion rate which involved adjustments every 5 min.

In Eq. 2 describing insulin dynamics, n_1_ is the insulin degradation constant, γ_1_ is a measure of insulin secretion and *ψ*(G[t]) is the dose-response relationship for glucose-dependent insulin secretion. Two models were used to describe insulin dynamics. The dose-response function, *ψ*(G[t]), is represented by a Hill function, Eq. 4a, in Model 1. While the Hill function has been used by other researchers, (Panunzi et al., 2007), the parameter K in Eq. 4 in this paper is fixed at the value obtained by fitting dose-response data from *in vitro* human pancreatic islet level studies (Walker et al., 2011) and is set at 17 mM.

**FIGURE 1:**
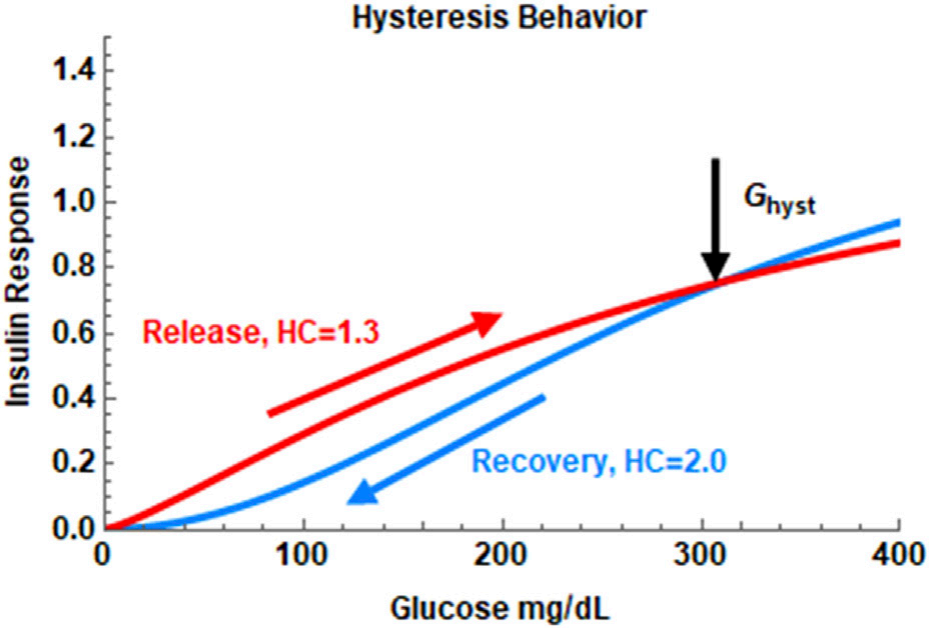
The hysteresis behavior of the insulin dose-response in Model 2. In this example, the Hill coefficient, h, is set at 1.3 during the insulin release phase and set at 2.0 during the recovery phase. The hysteresis turning point is set at the maximum of the glucose profile in the IIGI experiment.

As some researchers (Mari et al., 2002a; Keenan et al., 2012; Parekh et al., 2014) have raised the possibility of hysteresis-like behavior in insulin secretion in response to exogenous glucose influx, in Model 2, Eq. 4b was used to fit the IIGI data. In the hysteresis model, insulin secretory response to glucose depends on whether glucose levels are increasing or decreasing. The Hill coefficient h_1_ controls the response when glucose levels are increasing and h_2_ describes the secretory response when glucose levels are decreasing. C_1_ is an adjustment constant determined to make the two curves meet at the hysteresis point, (G_hyst_, t_hyst_). A sample plot showing hysteretic dose-response is shown in Figure 1. Here h_1_ is set to be lower than h_2_. Two Hill equations are used here as studies at the islet level indicate that the physiological dose-response shows this behavior. Logistic functions have been used in the paper by Keenan et al. to model hysteresis in c-peptide secretion. (Keenan et al., 2012). Changes in insulin secretory patterns with time have also been modeled using different potentiation factors as in the work by Mari et al. which is in turn derived from deconvolution of c-peptide kinetics. (Mari et al., 2002a).

Glucagon dynamics is described by Eq. 3 and is the sum of two terms, a clearance term, and a glucose dependent response term. Glucagon degradation or clearance is assumed to be first order in glucagon with degradation constant, n_2,_ which was obtained from the literature. (Alford et al., 1976). The second term describes the response to glucose. Islet level (Walker et al., 2011) and other studies (De Gaetano and Hardy, 2019) indicate that glucagon levels decrease exponentially as a function of glucose elevation. Preliminary investigations while modeling OGTT experiments showed that the glucagon dynamics shows hysteresis like behavior in response to glucose challenge. The suppression of glucagon in response to glucose challenge follows a different glucose dependence than the recovery after the plasma glucose level reaches a maximum. Thus, the glucagon dose-response is given by two different exponential terms (Eq. 5), one when glucose level is rising and a different one when glucose level is falling. The change in behavior is assumed to occur at the maximum of the glucose curve occurring at glucose concentration G_hyst_, and time t_hyst_. G_hyst_ is determined by finding the maximum of the plasma glucose profile, ie., the IIGI data, numerically and t_hyst_ is the time at which the maximum occurs (Wolfram Research, Inc, 2019). The reason for this slow recovery of glucagon levels post glucose influx is uncertain but likely due to paracrine modulation of glucagon secretion while the early suppression is likely due to intrinsic regulation by glucose. As both paracrine regulators, insulin and somatostatin, are secreted in a glucose dependent manner, here the paracrine modulation is also assumed to occur in a glucose dependent manner without explicit dependence on insulin or somatostatin concentration. The two exponential glucose dependent response terms were able to capture glucagon dynamics reasonably well during the 240 min duration of the IIGI experiment as shown in the results section. This persistent suppression of glucagon was also observed by Gerich (Mitrakou et al., 1990; Gerich, 1993) and in a larger study by Faerch et al. (Færch et al., 2016) The suppression and recovery constants are k_1_ and k_2_ respectively. The rate constant γ_2_ is a measure of glucagon secretion. The parameters τ, τ_1_ and τ_2_ represent possible time delays in glucose distribution, insulin secretion and glucagon suppression respectively.

$$\frac{dG\left[t\right]}{dt}=-\left(S_{G}+a_{1}I\left[t\right]\right)G\left[t\right]+a_{2}A\left[t\right]+R_{IIGI}\left[t-\tau \right]/V\;$$ (1)

$$\frac{dI\left[t\right]}{dt}=-n_{1}I\left(t\right)+\gamma_{1}\psi \left(G\left[t-\tau_{1}\right]\right)$$ (2)

$$\frac{dA\left[t\right]}{dt}=-n_{2}A\left(t\right)+\gamma_{2}\phi \left(G\left[t-\tau_{2}\right]\right)$$ (3)

$$\psi_{Hill}=\frac{1.5G{\left[t\right]}^{h}}{K^{h}+G{\left[t\right]}^{h}}\mathrm{(a)}$$

$$\psi_{Hysteresis}=\{\begin{array}{c} \frac{1.5G{\left[t\right]}^{h_{1}}}{K^{h_{1}}+G{\left[t\right]}^{h_{1}}},t<t_{hyst}\\ \frac{C_{1}G{\left[t\right]}^{h_{2}}}{K^{h_{2}}+G{\left[t\right]}^{h_{2}}},t\geq t_{hyst} \end{array}\mathrm{(b)}$$ (4)

$$\phi \left(G\left[t-\tau_{2}\right]\right)=\{\begin{array}{c} e^{-k_{1}G\left[t-t_{2}\right]},t<t_{hyst}\\ e^{-k_{2}G\left[t-t_{2}\right]}+yshift,t\geq t_{hyst} \end{array}$$ (5)

$$\mathrm{yshift}=e^{-k_{1}G_{\mathrm{hyst}}}$$

$$R_{IIGI}mg{\left(kg\;\min\right)}^{-1}=G_{Infusion}\left(g\right)/15\left(\min\right)\times \frac{1000}{\left(subject\;weight\;\left(kg\right)\right)}$$ (6)

### 2.2 Parameter estimation and statistics

Models 1 and 2 were simultaneously fit to glucose, insulin and glucagon data from IIGI tests on eight patients with diabetes (T2D) and eight weight matched control subjects (CS) without diabetes. (Bagger et al., 2011; Bagger et al., 2014). The glucose infusion in IIGI was manually adjusted to obtain a glucose profile that matches the OGTT glucose profile. The data available from the glucose infusion was the total amount of glucose infused in 15-min blocks for a total of 240 min. A uniform glucose infusion rate was thus used for every 15-min block of the infusion experiment as described in Eq. 6. This approximates the actual infusion rate which was adjusted every 5 min. As this approximation was applied across all patients, trends in estimated parameters within groups and between groups should likely be unaffected.

The parameters that were determined from the fit are glucagon action a_2_, secretion γ_2_ and suppression k_1_, insulin action a_1_, secretion γ_1_ and the Hill coefficients h, or h_1_ and h_2_ depending on the model used. As the exogenous glucose arrival, R_IIGI_ is continuous but not smooth, the time delay terms could not be estimated using the Levenberg-Marquardt algorithm in all subjects. The times delays, τ, τ_1_, τ_2_, were therefore adjusted manually. The glucagon recovery parameter k_2_ was also adjusted manually. These parameters were adjusted to obtain a reasonable visual fit before running the Levenberg-Marquardt algorithm to estimate the other parameters. The time delays as well as k_2_ were easy to set manually as good visual fits were obtained over a relatively narrow range of parameter values. No constraints were set on the values. The parameters n_1_, n_2_ and S_G_ were obtained from the literature and set at 0.14 min^−1^, (Duckworth et al., 1998), 0.08 min^−1^, (Alford et al., 1976; De Gaetano and Hardy, 2019; Grøndahl et al., 2021), and 0.014 min^−1^ (Dalla Man et al., 2002) respectively. V was fixed at 1.35 dL/kg. (Man et al., 2005).

The fitting was done using the nonlinear regression package NonLinearModelFit in Wolfram Mathematica, Version 12.0. (Wolfram Research, Inc, 2019). The Levenberg-Marquardt algorithm was used for the least-squares minimization. This package also provides all the statistics related to the fits.

A weighted least-squares regression was used for some of the subjects to improve the fits. The weights were determined using the coefficient of variation (CV) for glucose, insulin, and glucagon concentrations. The CVs used were 2%, 3% and 5.5% for glucose, insulin, and glucagon respectively. The caveat with using a constant CV in least squares fitting is that the fit is skewed heavily towards lower data values.

Significance of differences in parameters between groups (T2D vs. CS) was tested using the non-parametric Mann-Whitney *U* test. (MannWhitneyTest. Wolfram Research, 2010). The *p* values <0 .05 indicated significant differences between groups based on the null hypothesis that the median difference is zero. Correlations between parameters were determined using the nonparametric Spearman Rank Test. Comparison of Model 1 and Model 2 was done based on the Akaike Information Criterion corrected for small sample size (AICc). (Akaike, 1974; Portet, 2020).

Identifiability of parameters determined was checked using publicly available software, STRIKE-GOLDD Version 3.0. (Villaverde et al., 2016; Villaverde et al., 2019). All parameters in the model that were estimated using the least-squares fitting were assessed to be locally structurally identifiable.

Model validation (Hasdemir et al., 2015) was carried out by simulating data from IIGI experiments that matched OGTT glucose profiles with varying glucose loads (Bagger et al., 2014) on the same set of patients with T2D and CS as in this study. The results are presented in the supplementary section.

## 3 Experimental methods

The experimental methods are discussed in detail in the paper by Bagger et al. (Bagger et al., 2011; Bagger et al., 2014) A brief overview of the individuals and methods used is presented here.

### 3.1 Subjects

Eight patients (3 male) with T2D [mean age, 57 (range 40–75) years.; body mass index (BMI), 29 (25–34) kg/m^2^; duration of diabetes, 8 (6–36) months] and eight gender-, age-, and BMI-matched healthy control individuals [age, 57 (38–74) years.; BMI, 29 (26–33) kg/m^2^] were studied. All patients with T2D were diagnosed based on the criteria of the World Health Organization. (Expert Committee on the Diagnosis, 2003).

### 3.2 Experimental design

Participants were subject to OGTT followed by IIGI on a subsequent day. The subjects were studied in the morning in a recumbent position after an overnight fast (10 h) fast. On OGTT days, the participants ingested 75 g glucose dissolved in 300 g water. Blood samples were drawn 15, 10, 0 before and 5, 10, 15, 20, 25, 30, 35, 40, 45, 50, 60, 70, 90, 120, 150, 180, 240 min after ingestion of glucose. IIGI was performed using a sterile 20% wt/vol glucose infusion. The infusion rate was adjusted aiming at duplication of the plasma glucose profiles determined on the corresponding OGTT day. Blood was sampled as on the OGTT days. Analytical methods used to determine glucose, insulin and glucagon concentrations are described in Bagger et al. (Bagger et al., 2014).

## 4 Results

In the first and second subsections, the fits obtained using Model 1 for CS are discussed first, followed by the fits for patients with T2D, and trends within groups presented. In the third subsection, the parameters obtained for CS and patients with T2D are compared. In the fourth subsection correlations with hemoglobin A1c (HbA1c), fasting plasma glucose (FPG) and 2-h plasma glucose (2 h PG) will be presented and implications for categorizing patients with T2D in terms of impaired glucagon suppression in addition to insulin sensitivity will be discussed. In the fourth subsection the question of possible hysteresis behavior in glucose dependent insulin secretion will be explored by comparing Models 1 and 2 of insulin secretion. The implications with respect to postprandial glucose lowering will be discussed.

### 4.1 Control subjects

**FIGURE 2:**
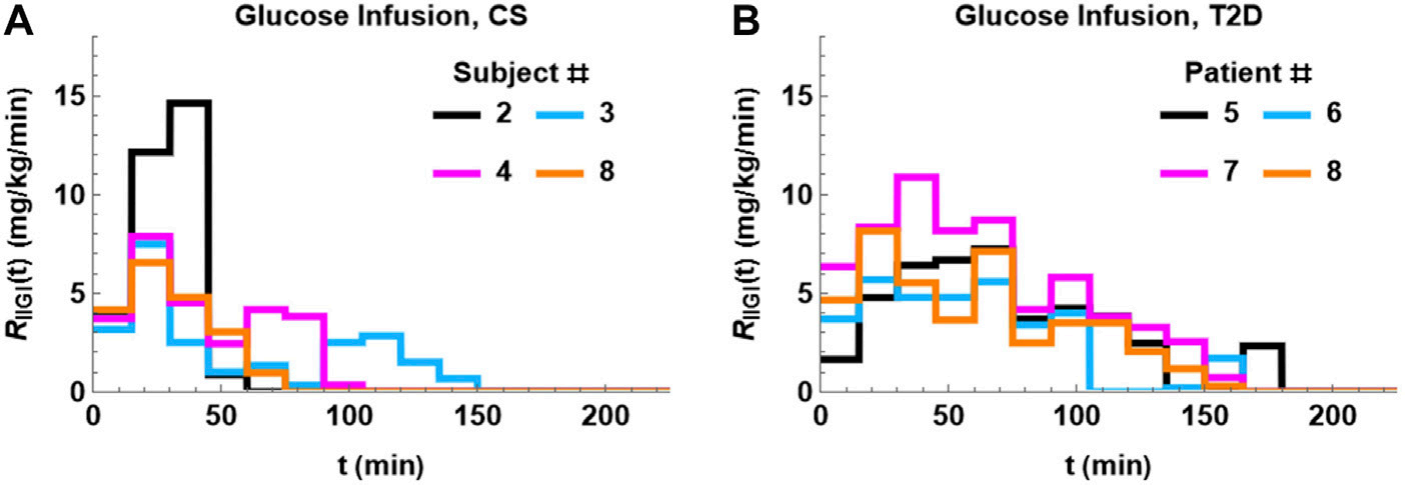
Glucose arrival profiles, R_IIGI_, determined using Eq. 6. There is significant difference between the profiles both within groups and between groups. The profiles tend be more bimodal in CS subjects **(A)**. The glucose arrival profile for T2D **(B)** is unimodal and more prolonged relative to CS.

The glucose infusion data was first converted to a plasma glucose arrival profile using Eq. 6. The glucose arrival rate profile in four CS subjects is plotted in Figure 2, panel A. This figure highlights the significant variation in glucose arrival profiles between the different subjects that could have an impact on extent of glucose excursions post glucose infusion. In addition, the shape of the plasma glucose profile in IIGI is also dictated by the shape of the exogenous glucose input, which in turn depends on the glucose excursions during the prior OGTTs.

**FIGURE 3:**
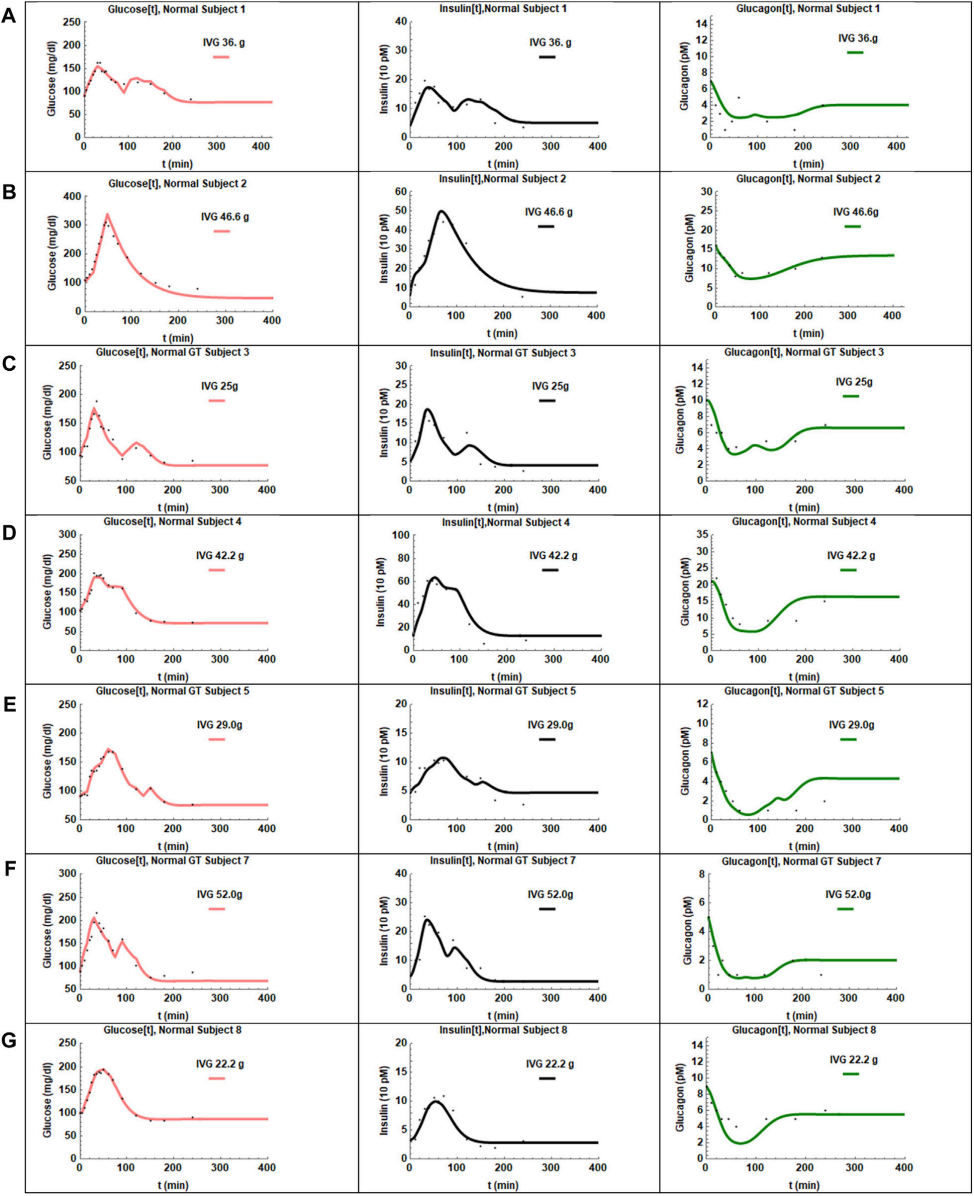
The fits obtained for the seven CS subjects are presented in panels **(A–G)**. The adjusted R^2^ values were >0.97 for all the fits. The glucose profiles were fit the best by the model. In some cases, the insulin recovery was not captured perfectly **(D,E)**. Glucagon shows very slow recovery and is captured reasonably well by the hysteresis model except for one subject **(E)**.

The coupled Eqs 1–3 with insulin response given by Eq. 4a) were then fit to the data. A reasonable fit with low standard errors was not obtained for CS 6. The IIGI experiments involve manual adjustment of glucose infusion rates to obtain glucose profiles that match the OGTT profiles which sometimes result in overshoot in plasma glucose values that may trigger first phase insulin secretion which is likely what happened in CS 6 and could not be fit with this model. This subject was excluded from further study. The fits obtained for the remaining seven CS subjects are presented in Figure 3 and the estimated and manually adjusted parameters in Table 1. The fits were uniformly good for CS subjects with high coefficient of determination (adjusted *R* ^2^) values >0 .97. The standard errors in all the estimated parameters were low and the *p*-values for all the parameters <0.05 except for a_1_ of patient 2. The average values, standard errors of the mean and ranges of the parameters are presented in Table 3.

**TABLE 1:** Parameters obtained from fitting the coupled Model 1 to the seven CS subjects.

CS#	Parameter								
	a_1_	a_2_	*γ*_1_	*γ*_2_	k_1_	k_2_	h	*τ*	*τ*_*1*_
1	0.00061	0.33	8.7	1.7	0.25	0.55	2.0	-	-
2	0.000055	0.052	9.3	1.3	0.046	0.35	1.4	3.0	12
3	0.00071	0.20	8.7	3.6	0.27	0.55	2.2	5.3	-
4	0.00012	0.068	21	8.1	0.26	0.45	1.9	-	-
5	0.00032	0.28	3.2	3.4	0.48	0.55	1.3	-	-
7	0.0012	0.59	8.9	0.92	0.24	0.50	2.3	-	-
8	0.00063	0.25	3.2	2.9	0.28	0.55	1.9	-	-

The CS subjects showed a wide range of insulin sensitivities, a_1_, 0.000055–0.0012. Subjects two and four showed lower insulin sensitivity relative to other CS subjects. The range in insulin sensitivities can be attributed to the fact that the CS subjects were weight matched to the T2D group (average BMI = 29). The glucose dependent insulin secretion parameter γ_1_ showed modest variation with one outlier, CS subject 4. The Hill coefficient, h, was divided into two groups, one centered around h = 2.0 and another around h = 1.35. Subjects with higher values of h have a steeper insulin dose-response to glucose. In general, there were no time delays in the CS subjects with respect to insulin secretion or action except for a 12-min delay in insulin secretion in CS 2 who also had the lowest insulin sensitivity.

The glucagon suppression parameter k_1_ in CS subjects was clustered around 0.26, close to the value of 0.25 determined from human islet level studies. Only one subject, CS 2, had anomalously low glucagon suppression. The average glucagon recovery parameter k_2_ was 0.50 and shows that glucagon recovery is much slower than suppression in CS subjects. The glucagon action parameter, a_2_, which is a measure of glucagon effectiveness in glucose release appeared to be significantly attenuated in CS 2 and 4 relative to the others; the average value was determined to be 0.25. The glucagon secretion parameter γ_2_ in normal subjects did not show a huge spread except for CS 4 who had a much higher value relative to others. No time delays, τ_2_, were observed in glucagon suppression.

There was also a short time delay, τ, in R_IIGI_ in CS subjects two and three of 3 and 5 min respectively. This behavior was observed in most patients with T2D.

### 4.2 Patients with T2D

**FIGURE 4:**
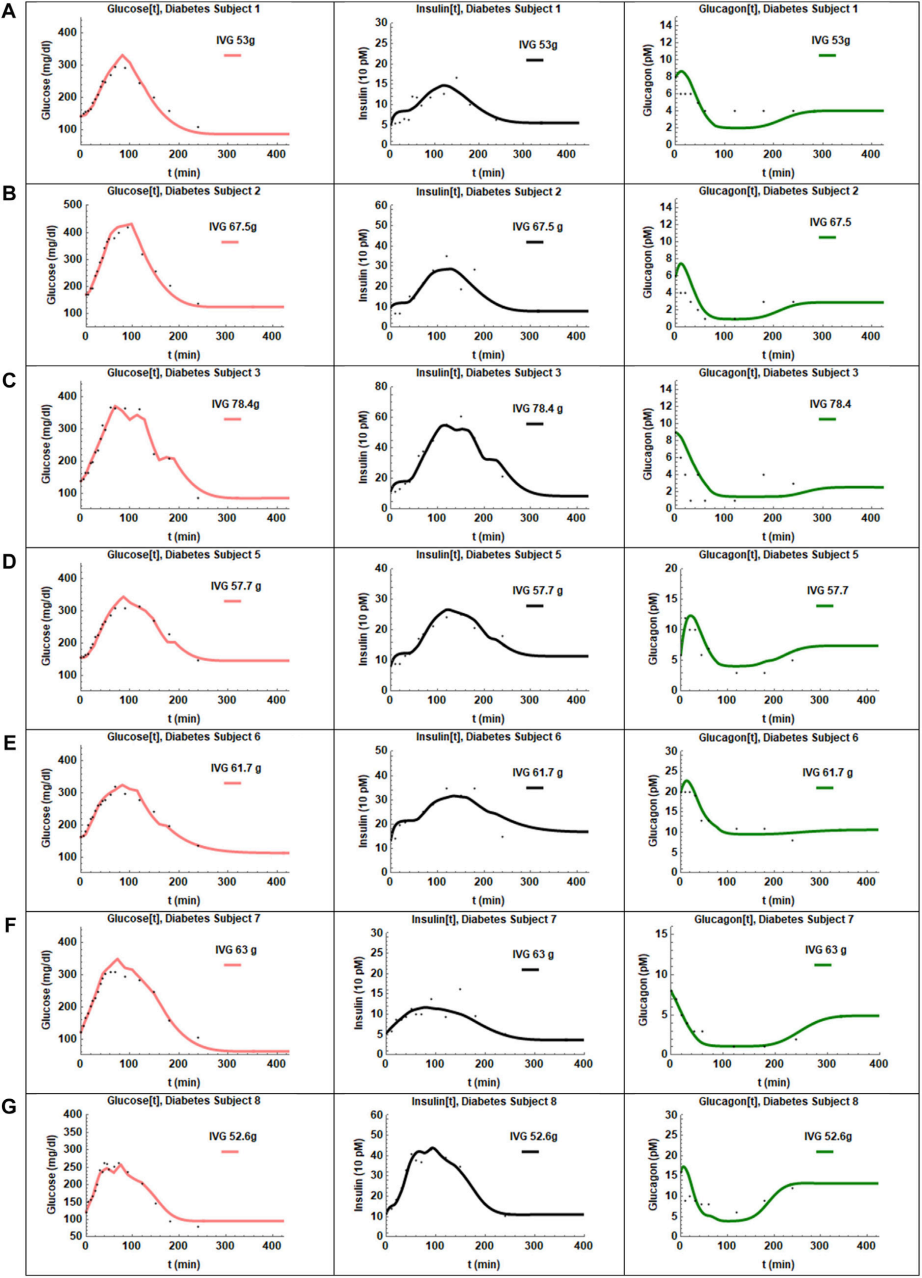
The fits obtained for the seven patients with T2D are presented in panels **(A–G)**. The adjusted R^2^ values were >0.98 for all subjects. The glucose and insulin profiles were fit best by the model. Glucagon recovery is slow as seen in panels **(A–G)** and again described reasonably well by the hysteresis model.

The coupled Eqs. 1– 3 with the plasma glucose arrival profile determined using Eq. 6 were fit to the data. The fits for the patients with T2D are shown in Figure 4 and the estimated parameters in Table 2. The adjusted *R* ^2^ values for the fit were high for all patients (>0.98). The SE values were low and *p* values were <0.05 for all parameters except for the insulin sensitivity parameter a_1_ in three patients. The fits for those three patients were particularly sensitive to initial guess values for the nonlinear least-squares regression. Possible reasons for the difficulty in fitting these patients might be: 1) The baseline glucagon data for subjects one and two did not match the values on OGTT day indicating greater uncertainty in glucagon data points. 2) T2D subject three had multiple data values near the detection limit of glucagon. The average values, standard errors of the mean and ranges of the parameters are presented in Table 3. Of the eight patients studied patient four could not be fit, likely due to the overshoot in infused glucose as described previously and was excluded from further study.

**TABLE 2:** Parameters obtained from fitting the coupled Model 1 to patients with T2D.

T2D#	Parameter								
	a_1_	a_2_	*γ*_*1*_	*γ*_*2*_	k_1_	k_2_	h	*τ*	*τ*_*1*_
1	0.00010	0.30	2.7	2.8	0.17	0.60	1.1	10	30
2	0.000085	0.50	4.2	3.6	0.17	0.45	1.7	8	30
3	0.000094	0.49	8.9	1.9	0.13	0.65	1.8	10	35
5	0.000072	0.29	4.6	4.0	0.14	0.33	1.6	12	30
6	0.000050	0.16	5.8	5.2	0.10	0.65	1.0	10	45
7	0.00011	0.18	2.0	1.4	0.16	0.45	1.0	-	-
8	0.000080	0.11	10	8.6	0.23	0.45	1.9	-	15

**TABLE 3:** Average values, standard errors, and ranges of the parameters in patients with T2D and CS subjects.

ACT	Mean		SEM		Range	
	T2D	CS	T2D	CS	T2D	CS
a_1_ (×10^−5^)						
(10 pM min)^−1^	8.4	52	0.75	15	5.-11	5.5–120
a_2_ mg/dL (pM min)^−1^	0.29	0.25	0.059	0.069	0.11–0.5	0.052–0.59
γ_1_ 10 pM min^−1^	5.5	9.0	1.1	2.2	2.0–10	3.2–21
γ_2_ pM min^−1^	3.9	3.1	0.92	0.92	1.4–8.6	0.92–8.12
k_1_ (mM)^−1^	0.16	0.26	0.015	0.047	0.1–0.23	0.046–0.48
k_2_ (mM)^−1^	0.51	0.50	0.046	0.029	0.33–0.60	0.35–0.55
h	1.4	1.9	0.15	0.14	1.0–1.9	1.3–2.3
τ_1_ (min)^−1^	26	1.7	5.5	1.7	0.-45	0.-12
τ (min)^−1^	7.1	1.2	1.9	0.80	0.-12	3.0–5.3

Patients with T2D showed a large delay in insulin secretion, τ_1_, with a mean value of 26 min. The insulin sensitivities, a_1_, in patients with T2D were narrowly distributed around the mean value of 0.000084. The insulin secretion parameter, γ_1_, had an average of value of 5.5 and a narrow spread of 1.1. The Hill coefficient clustered around two values, four patients around h = 1.75 and three patients around h = 1.0.

The glucagon suppression parameter in patients with T2D was below 0.2 for all subjects except subject 8. Values of glucagon action, a_2_, were mixed with four subjects showing significantly higher values than the other three. The glucagon secretion parameters, γ_2_, were evenly distributed about the mean except for subject 8. A time delay, τ_2_, of 5 minutes was observed in one patient.

The glucose arrival rate R_IIGI_ profile in four patients with T2D is plotted in Figure 2, panel B. The infusion profiles are unimodal and prolonged, extending to 180 min in some subjects. The infusion profiles in T2D show less variability than the CS subjects. Remarkably, a time delay, τ, had to be introduced in R_IIGI_ and had an average of 7 min with 12 min being the longest delay.

### 4.3 Comparison of patients with T2D and CS subjects

Mean values and ranges of the parameters for patients with T2D and CS subjects are presented in Table 3. The Mann-Whitney *U* test was used to compare the median differences between T2D and CS parameters. The results of the comparison test are presented in Table 4. There are significant differences (*p*-value<0.05) between five of the T2D and CS parameters, namely: the insulin sensitivity parameter, a_1_, the glucagon suppression parameter k_1_, the Hill coefficient, h, in the insulin dose-response curve and the time delays in insulin secretion and exogenous glucose arrival.

**TABLE 4:** Results of the Mann-Whitney *U* test. The insulin sensitivity parameter, a_1_, the glucagon suppression parameter k_1_, the Hill coefficient h, the insulin secretion time delay τ_1_ and the infused glucose R_IIGI_ time delay τ, are found to be significantly different between the T2D and CS groups.

Parameter	Mann-whitney *U* test (T2D vs. CS) *p*-value
a_1_	0.015
a_2_	0.70
γ_1_	0.28
γ_2_	0.37
k_1_	0.021
k_2_	0.95
h	0.039
τ	0.006
τ_1_	0.039

Insulin sensitivity, a_1_, is much lower in patients with T2D than CS subjects except for two outliers CS 2 and four who had insulin sensitivities on par with patients with T2D. Homa-IR (84) is a method of estimating insulin resistance from fasting glucose and insulin levels. The insulin sensitivity parameters determined using Model 1 showed a positive correlation with 1/HomaIR (*n* = 14 (CS and T2D patients), Spearman Rank Test correlation = 0.70, *p* value = .0056). The Hill coefficient, h, which is a measure of the rapidity of the insulin response to glucose elevation, is higher in CS subjects than T2D subjects. Insulin secretion is delayed on average by 26 min in patients with T2D. In addition, there was delay of ∼10 min in R_IIGI_ circulation in patients with T2D.

The glucagon suppression constant k_1_ is significantly lower in patients with T2D relative to CS. Glucagon action in the liver is not significantly different between CS and T2D though there is significant variation within groups. The glucagon secretion parameter is not significantly different between T2D and CS groups in this study.

The glucose infusion profiles are also different between the two groups. The infusion profiles of the CS subjects are bimodal (two peaks), shorter, and show more variability relative to patients with T2D. The infusion profile in patients with T2D is unimodal and more prolonged lasting up to 180 min in some cases.

#### 4.3.1 Trends in parameters

**FIGURE 5:**
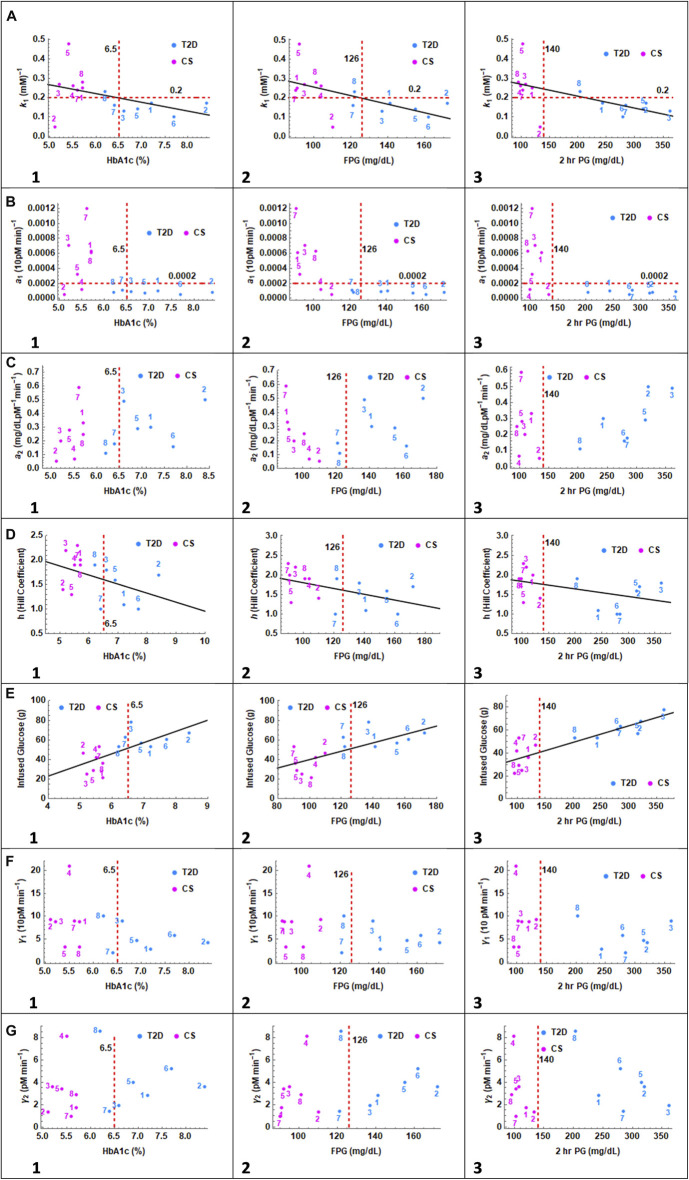
In the series of panels **(A)** through **(G)**, the parameters related to glucagon suppression k_1_, insulin action a_1_, glucagon action a_2_, Hill coefficient h, infused glucose, insulin secretion γ_1_ and glucagon secretion γ_2_ are plotted as a function of HbA1c, FPG and 2 h PG values. In panel **(A)** 1, 2, and 3, CS and T2D subjects partition into distinct quadrants except for one CS subject. A linear relationship is observed between glucagon suppression and HbA1c, FPG, and 2 h PG. The cutoff value separating patients with T2D, and CS subjects is set at 0.2. In Panel **(B)**, the correlation with insulin sensitivity parameter a_1_ is presented. CS subjects and patients with T2D again partition into two distinct quadrants except for CS 2 and 4. The cutoff value separating patients with T2D, and CS subjects is 0.0002. In panel **(C)** no clear distinction between patients with T2D and CS subjects is observed with respect to glucagon action parameter a_2_. In Panel **(D)**, the Hill coefficient, h, is trending higher in CS subjects toward h = two and in patients with T2D towards h = 1. In Panel **(E)**, infused glucose, an indicator of the incretin effect, shows a linear trend with respect to HbA1C, FPG and 2 h PG, particularly in patients with T2D. In panel **(F)**, no difference in insulin secretion parameter, γ_1_, between CS and T2D is observed. In panel **(G)**, patients with T2D show a weak linear trend towards higher HbA1c and FPG values with increasing glucagon secretion parameter.

Fasting plasma glucose (FPG), HbA1c and 2 h OGTT plasma glucose levels are all used to diagnose diabetes. (Diabetes Association, 2022). The parameters obtained from the fits were plotted against FPG, HbA1c and 2 h PG (which is approximately matched to 2 h OGTT glucose) to see which parameters correlated with these determinants of diabetes. The contribution of parameters related to glucagon dynamics to impaired FPG, HbA1c and 2 h PG levels is established. As this model differs significantly from previously established models, parameters related to insulin sensitivity and dose-response are classified based on correlations with FPG, HbA1c and 2 h PG. As the sample size is small, the cut-off valus separating T2D and CS are tentatively assigned by visual inspection of Figure 5 and not through statistical tools such as receiver operating characteristic (ROC) curves.

In panel A, the glucagon suppression parameter, k_1_, is plotted against HbA1c, FPG and 2 h PG. A linear trend was observed in all three cases with glucose dependent glucagon suppression constant decreasing with increasing A1c, FG and 2 h PG. Two outliers are observed, CS subject 5 with very high suppression and CS subject 2 with very low suppression. The patients with T2D and CS subjects separate into two distinct non-overlapping quadrants, particularly when plotted against 2 h PG. The k_1_ value of 0.2 serves as the demarcation between CS and T2D groups. The Spearman Rank Correlation Coefficient and the *p*-value between k_1_ and HbA1c, FPG, and 2 h PG with and without the two outliers are: (*n* = 14; (−0.46,0.1), (−0.67,.009), (−0.75, 0.002)), and (*n* = 12; (−0.79, 0.002), (−0.73,.007), (−0.86,.0003)) respectively.

In panel B, the insulin sensitivity parameter, a_1,_ is plotted against HbA1c, FPG and 2 h PG. All patients with T2D show uniformly low values of insulin sensitivity and again fall into a separate quadrant. Two CS subjects overlap with patients with T2D with respect to a_1_. The demarcation for a_1_ values between T2D and CS is set at 0.0002 which would place CS 2 and 4 in the diabetes group.

In panel C the glucagon action parameter, a_2,_ is plotted against HbA1c, FPG and 2 h PG. The action parameter is a measure of glucagon dependent glucose release from the liver. The glucagon action parameter is not significantly different between the two groups. There are no clear trends with respect to a_2_ though within the T2D group, 2 h PG values increase linearly with increasing a_2_.

In panel D the Hill coefficient which describes the steepness of the glucose dependent insulin response is plotted against HbA1c, FPG and 2 h PG. The Hill coefficient decreases with increasing HbA1c and FPG levels. A value of h = 2 appears to be closer to normal secretory response and h = 1 appears to be the low end of the response.

In Panel E, the total glucose infused in the IIGI experiment is plotted against HbA1c, FPG and 2 h PG. The premise of the IIGI experiment is that if the insulin response to glucose challenge is entirely glucose dependent, with no incretin effect, then the amount of glucose infused will be identical to that of the OGTT glucose challenge experiment. In this case it would be 75 g glucose as in the matching OGTT. The lower the amount of glucose required, the greater the incretin effect. (Nauck et al., 1986). A strong linear relationship is seen with 2 h PG levels particularly within the T2D group. The T2D patient three who showed no incretin effect, had the highest 2 h PG level. The Spearman Rank Correlation Coefficient and the *p*-value between Glucose_Infused_ and HbA1c, FPG, and 2 h PG are (0.74,.0025), (0.72,.0035), and (0.88,.000039) respectively.

In Panel F insulin secretion parameter γ_1_ is presented. There is no difference between the insulin secretion parameter between the T2D and CS groups and no trends with respect to HbA1c, FPG or 2 h PG. This implies that incretin effects are primarily responsible for differences in insulin secretion between the two groups under OGTT conditions.

In Panel G, the glucagon secretion parameter γ_2_ is plotted as a function of HbA1C, FPG and 2 h PG. There is trend towards increasing HbA1c and fasting glucose with increasing glucagon secretion within the T2D group. No pattern was seen with respect to 2 h glucose.

### 4.4 Hysteresis in insulin secretion

To assess the role of hysteresis in glucose dependent insulin secretion, Model 1 without hysteresis, Eq. 4a was compared with Model 2, where insulin secretion is described by Eq. 4b. In Model 1, the insulin secretion is described by a single Hill function with coefficient h. In the hysteresis model, insulin secretion is described by two Hill functions with coefficients h_1_ and h_2_. The Hill coefficients h_1_ and h_2_ are varied between the rising and recovery phases of insulin secretion, but the parameter K is assumed to be constant and same in both models.

The results in Section 4, describe the fits obtained with Model 1 for CS and T2D subjects. The parameters obtained using the hysteresis Model 2 are presented in Tables 5, 6. The fits are presented in Supplementary Figures S3, 4. Visually the fits for the different subjects are not very different for Model 1 and Model 2. In Model 2, the Hill coefficients h_2_ is greater than h_1_ for all CS subjects. Thus, insulin levels fall more steeply when glucose is declining. In the patients with T2D h_1_ ∼ h_2_ indicating there is no hysteresis and the results are essentially equivalent to the Model 1. The values of h in Model 1 were comparable to h_2_ in Model 2 in CS subjects. In patients with T2D, h was comparable to h_1_ and h_2_, in other words, a single Hill equation is sufficient to describe insulin dose-response.

**TABLE 5:** Parameters determined from fitting the hysteresis Model 2 to data from CS subjects. The parameters obtained are similar to that for Hill Model 1. The values of h_1_ are lower than h_2_ in all subjects implying a steeper glucose dependent dose-response during the insulin recovery phase.

CS#	a_1_	a_2_	*γ*_*1*_	*γ*_*2*_	k_1_	k_2_	h_1_	h_2_	*τ*_*1*_ */τ*
1	0.00053	0.34	6.0	2.9	0.35	0.55	1.4	1.8	−/−
2	0.000058	0.092	9.0	2.0	0.077	0.35	1.3	1.8	14/3.5
3	0.00062	0.18	5.7	4.7	0.31	0.55	1.4	2.3	6./5
4	0.00011	0.070	16	8.8	0.28	0.55	1.1	2.2	−/−
5	0.00034	0.28	2.7	3.3	0.46	0.45	0.94	1.4	−/−
7	0.0011	0.58	8.6	0.96	0.24	0.55	2.2	2.4	−/−
8	0.00063	0.22	2.7	2.9	0.25	0.45	1.3	1.7	−/−

**TABLE 6:** Parameters determined from fitting the hysteresis Model 2 to data from patients with T2D. The parameters a_1_-k_2_ are comparable to that for Hill Model 1. The value of h_1_ is approximately equal to h_2_ in all subjects implying there is no hysteresis in diabetic subjects. Only subject eight showed a significant drop in h_2_ compared to h_1_.

T2D#	a_1_	a_2_	*γ*_*1*_	*γ*_*2*_	k_1_	k_2_	h_1_	h_2_	*τ*_*1*_ */τ*
1	0.000092	0.33	2.6	2.5	0.17	0.4	1.2	1.3	30/10
2	0.000075	0.57	4.5	3.6	0.18	0.35	1.4	2.0	30/8
3	0.000081	0.45	9.2	2.1	0.13	0.65	2.3	2.0	30/10
5	0.000072	0.26	4.5	4.5	0.14	0.41	1.5	1.4	30/12
6	0.0001	0.19	5.4	4.2	0.097	0.65	0.9	0.95	45/10
7	0.00011	0.18	2.0	1.1	0.15	0.45	1.1	0.90	−/−
8	0.00011	0.12	10	5.1	0.18	0.3	2.2	1.2	15/-

In Model 1, h was significantly higher in CS subjects compared to patients with T2D. In Model 2, h_2_ is significantly higher in CS subjects relative to patients with T2D. So, both models show a change in behaviour in patients with T2D.

Model comparison is made based on the Akaike Information Criterion with small sample correction (AICc). (Akaike, 1974). This criterion gives an estimate of whether the model with more parameters reduces the error sufficiently to justify the increase in complexity. The Akaike criterion can only be used to compare models using the same data set, so the AICc values are presented in Tables 7, 8 for individual CS and T2D subjects respectively. The lower the AICc value, the better the fit. The differences in AICc values are given in column 4. The AICc differences were variable with some subjects fit better by Model 1 and others by Model 2. The criterion cannot therefore be used to pick one model over the other.

**TABLE 7:** Values of the Akaike Information Criterion corrected for small sample size (AICc) is presented for patients with T2D. Smaller AICc values indicate a better fit. The AICc values are not uniformly less for one model over the other in all patients.

Pat#(T2D)	Akaike information criterion (AICc)		AICc difference (model 1-Model2)
	Model 1 (Hill)	Model 2 (hysteresis)	
1	309	303	+6
2	318	317	+1
3	321	323	−2
5	296	301	−5
6	290	292	−2
7	232	233	−1
8	301	297	+4

**TABLE 8:** Values of the Akaike Information Criterion corrected for small sample size (AICc) is presented for CS subjects. Smaller AICc values indicate a better fit. The AICc values are not uniformly less for one model over the other.

Pat#(CS)	Akaike information criterion (AICc)		AICc difference
Model 1-model 2	Model 1 (Hill)	Model 2 (hysteresis)	
1	256	257	−1
2	299	305	−6
3	236	227	+9
5	279	268	+11
6	229	231	−2
7	253	256	−3
8	217	216	+1

## 5 Discussion

T2D is a disease that is manifested when insulin resistance and beta- and alpha-cell dysfunction occur. (Topp et al., 2000; Topp et al., 2007; Burcelin et al., 2008; Ashcroft and Rorsman, 2012; Ha et al., 2016). As these three determinants of diabetes are intrinsically coupled, it is important to quantify parameters related to them in a self-consistent manner without splitting the coupled dynamics into separate subsystems. The parsimonious coupled system of delay differential equations used in this paper allow for estimation of all parameters in a single step. The coupled glucose-insulin-glucagon model was used to fit data from IIGI experiments to quantify glucagon action, suppression, and secretion as well as insulin resistance and secretion, without the confounding influence of incretins and other gut mediated factors. The results presented in Section 4 show that the model captures the coupled dynamics correctly and yields parameters related to both alpha and beta cell dysfunction and insulin resistance in one step. As this is a new extended model based on delay differential equations, some comparisons will be made with parameters related to insulin resistance and secretion from the single delay differential model of De Gaetano et al., where the coupled insulin-glucose dynamics was studied. (Panunzi et al., 2007; De Gaetano and Hardy, 2019).

Alpha cell dysfunction is known to contribute to both fasting and postprandial hyperglycemia in T2D. (Dunning and Gerich, 2007; Burcelin et al., 2008; Lund et al., 2014). Increased glucagon secretion, lowered glucagon suppression and differences in glucagon action could contribute to elevated fasting glucose levels and continued glucose production in the post prandial state. In this study, the glucagon suppression parameter, k_1_, was found to be significantly lower in patients with T2D relative to CS. The parameter also showed clear linear relationship with respect to HbA1c, FPG and 2 h PG values. There was strong negative correlation with all three indicators of diabetes. HbA1c levels have been shown to correlate better with post-prandial glucose levels and less with fasting glucose levels. (Landgraf, 2004; Hershon et al., 2019). Two hr PG values are reflective of postprandial glucose excursions. This shows that glucagon suppression is impaired in T2D and has an impact on both fasting and postprandial glucose levels and likely exacerbates hyperglycemia in patients with T2D.

The glucagon secretion parameter γ_2_ was not significantly different between CS and patients with T2D in this study. In the paper by Unger et al. (1970) similarly, statistically significant differences were not observed in fasting glucagon levels between CS and T2D subjects but when hyperglycemia was induced by glucose infusion in the CS so as to simulate the fasting hyperglycemia of T2D patients, mean glucagon fell significantly below the T2D mean, indicating the level of glucagonemia is high for the prevailing glycemia in T2D. This is also in line with former observations measuring hepatic glucose output using radiolabeled isotopes showing a clear positive correlation between baseline glucose and hepatic glucose output. (Baron et al., 1987). Even with great basal variation in basal glucagon the hepatic glucose output was suppressible by suppressing glucagon alone in pancreatic clamp (using somatostatin and basal insulin infusion). (Baron et al., 1987).

The glucagon action parameter, a_2_, which is a measure of how effective it is in hepatic glucose production, is not significantly different between the CS and T2D subjects and is thus not the likely cause of elevated fasting and post prandial plasma glucose levels.

Modeling IIGI gives information regarding glucose stimulated insulin secretion. In model 1, there are three parameters describing insulin secretion: 1) a measure of the magnitude of insulin secretion, *γ*_1_. 2) the steepness of the response based on the Hill coefficient, h, in the dose-response expression, *ψ* and 3) the time delay in insulin response, *τ*_1_. A point to note is that differences in hepatic insulin extraction (HPE) may exist between subjects and the insulin secretion parameters determined are reflective of post HPE plasma insulin levels. The differences in HPE could also account for the some of the variation in plasma insulin levels between subjects but is not considered here. (Bojsen-Møller et al., 2018; Santoleri and Titchenell, 2019; Piccinini and Bergman, 2020).

The parameter *γ*_1_ which is a measure of glucose dependent insulin secretion was not significantly different between T2D and CS subjects. There may be multiple reasons for this observation. The patients with T2D in this study were newly diagnosed and thus in the early stages of disease progression. This result is also consistent with the estimation of the incretin effects from the IIGI experiments in this study which showed that the incretin dependent insulin response is the dominant factor in differentiating between the levels of insulin secretion in CS and T2D subjects. The incretin dependent insulin secretion was found to be significantly impaired in patients with T2D. (Bagger et al., 2011).

Though the insulin secretion parameter was not significantly different, the steepness of the insulin response as reflected by the Hill coefficient, h, is significantly different between the two groups. A value of h = 2 appears to be closer to normal secretory response, observed in CS subjects with higher insulin sensitivity and h = 1 appears to be the low end of the response observed in T2D subjects who have much lower insulin sensitivity. A point of note is that the CS subjects in this study were weight matched to the patients with T2D and the T2D patients were newly diagnosed. It is possible that a clearer demarcation between CS and T2D groups with respect to the Hill coefficient might become evident when studying a wider spectrum of T2D and CS subjects. In the paper by De Gaetano et al., the average estimated value for the Hill coefficient in normal individuals was 2.4.

Significant differences in time delay in glucose stimulated insulin secretion, τ_1_, was observed between CS and T2D subjects. There was a significant time delay in only one CS subject who also had low insulin sensitivity whereas most T2D had large time delays in insulin secretion. The reason for the delay in insulin secretion is unclear but might be partly related to the delay in exogenous glucose (R_IIVG_) arrival observed in the patients with T2D. In the paper by De Gaetano where they fit data from IVGTT on normal subjects, a delay in the insulin secretion term had to be introduced to produce the characteristic second phase insulin secretion profile. This result is very different from that observed in this IIGI study where no significant delays were observed in glucose stimulated insulin secretion in the CS subjects.

The insulin sensitivity parameter showed significant differences between T2D and CS subjects following established trends. The magnitude of the average insulin sensitivity of 0.0005 (10 pM min)^−1^ in CS subjects is near the lower end of the glucose sensitivity parameter estimates in normal subjects in the paper by De Gaetano et al. In addition, two CS subjects had insulin sensitivities that were on par with T2D patients. This is likely because the CS group was weight matched to the patients with T2D in this study. In fact, some CS subjects showed very high levels of insulin secretion indicative of the compensatory phase in response to falling insulin sensitivities. A cut-off value of 0.0002 (10 pM min)^−1^ separating T2D and CS was tentatively assigned though a much larger study would be required to correctly identify the cut-off based on ROC curves for example. The insulin sensitivity measures determined in this study correlated well with HOMA-IR values. Though HOMA-IR is considered to be a measure of hepatic insulin resistance it has been found to correlate well with insulin sensitivity measures from the hyperinsulinemic-euglycemic clamp, for example. (Matthews et al., 1985).

**FIGURE 6:**
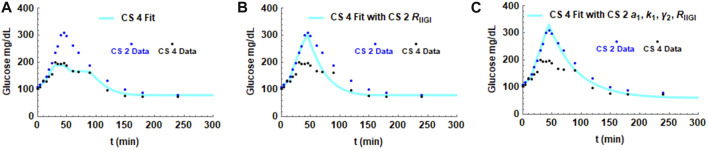
Simulations showing the effect of the exogenous glucose arrival R_IIGI_ on the glucose profile. Parameters in the fit for CS 4 were substituted with the values obtained from the CS 2 fit. Panel **(A)** shows the fit with all CS 4 parameters. Panel **(B)** shows the effect of substituting CS 2 glucose infusion, R_IIGI,_ in the simulation. Panel **(C)** shows the impact of substituting CS 2 insulin sensitivity parameter a_1_, glucagon secretion parameter k_1_ and glucagon secretion parameter γ_2_ in addition to R_IIGI_ on the CS 4 fit.

As seen in Figure 2, the exogenous glucose that is infused has distinct profiles for the different subjects that is particularly apparent in CS patients. To delineate the influence of infused glucose on post infusion glucose profiles, the effect of substituting R_IIGI_ of one patient with that of another was studied. In order to make a meaningful inference, two CS subjects, 2 and 4, who had similar parameters including similar amounts of total glucose infused (Table 1; Figure 3 Panel B and D), but showed very different post infusion glucose profiles were chosen for the simulations. In Figure 6A, the fit obtained for CS 4 (light blue solid line) as well as the glucose data for CS 2 and CS 4 are shown. In the second simulation (Figure 6B), all parameters of the CS 4 fit were retained but the exogenous glucose infusion R_IIGI_ of CS 2 was substituted. This causes the CS 4 glucose profile to spike very much like that seen in CS 2. In Panel C, 4 parameters of CS 2 were substituted retaining only insulin secretion and glucagon action parameters. It is shown that patient four transitions to patient two completely. The effect of the exogenous glucose profile is dramatic in this case. This result indicates that rate of glucose arrival could have a big impact on glucose dynamics. As IIGI is isoglycemic with the corresponding OGTT this suggests that rate of glucose arrival from the gut could play a role in glucose dynamics post oral ingestion as well and account in part for the differences in glucose excursions between subjects. The role of gastric emptying in glucose homeostasis has been studied by several researchers where this effect has been observed, eg., Holst et al. and references therein. (Brener et al., 1983; Horowitz et al., 1993; Holst et al., 2016). This possibility has been suggested in the paper by Fiorentino et al. where the role of sodium-glucose co-transporters is investigated. (Fiorentino et al., 2017). This result may also have direct relevance to the findings in the paper by Utzschneider et al. where they made an association between plasma glucose profile shape and beta cell function in newly diagnosed T2D patients. (Utzschneider et al., 2021).

A consequence of ingesting large glucose loads is often a lowering of glucose to values below baseline levels or postprandial hypoglycemia. (Saha, 2006; Parekh et al., 2014). This phenomenon is seen in most of the subjects in this study, particularly the CS subjects. One explanation could be that delayed recovery of glucagon to baseline levels causes the glucose levels in turn to fall below baseline. In the paper by Wang, G., (Wang, 2014)., hysteresis in insulin action is hypothesized to cause postprandial hypoglycemia. As modeling in this study with constant insulin action, a_1_, was able to reproduce the plasma glucose profiles correctly, including the postprandial dip, hysteresis behaviour in insulin secretion was considered a possibility instead. If insulin secretion falls off more slowly after glucose levels start falling, it could contribute to post-prandial lowering of glucose below baseline. The fits of the hysteresis model 2, showing h_1_<h_2_ in CS subjects, on the contrary, predict insulin levels returning to baseline levels more sharply than the rise. The hysteresis model of glucose dependent insulin secretion thus does not appear to explain post-prandial hypoglycemia. Secondly, the hysteresis model 2, with one extra parameter, did not provide a significantly improved description of the dynamics relative to the Hill model 1 as indicated by the AICc criterion.

Modeling IIGI is shown to reveal different levels of impairment in alpha- and beta-cell function and insulin action in T2D. The contribution of various parameters to glucose homeostasis, particularly those related to glucagon dynamics have been estimated. Quantification of the significant impairment in glucagon suppression in patients with T2D should help in classifying patients based on alpha-cell dysregulation. Changes in insulin dose-response parameters in T2D without the confounding influence of incretins and other gut mediated factors as well as first phase insulin release have been determined. The importance of considering exogenous glucose arrival on exacerbating postprandial glucose excursions is highlighted using model simulations. In addition to T2D, the model developed was also used to explore the role of hysteresis in insulin secretion in explaining phenomena such as post prandial glucose lowering and a related pathophysiology reactive hypoglycemia. Results from this study show that hysteresis in insulin secretion is not the likely cause of postprandial glucose lowering. While the model developed is shown to be very effective in determining parameters related to the coupled dynamics from IIGI data, shortcomings of fitting IIGI data are that some of the parameters had to be adjusted manually. Future work would include fitting the model to larger sets of data which would allow for classification of patients based on cut-off values of parameters related to both alpha- and beta-cell impairment determined from ROC curves.

## Data Availability

The original contributions presented in the study are included in the article/Supplementary Material, further inquiries can be directed to the corresponding authors.
