# Supplementary material for: A glucose-insulin-glucagon coupled model of the isoglycemic intravenous glucose infusion experiment
Source: Front Physiol. 2022 Sep 6;13:911616. doi: 10.3389/fphys.2022.911616 (PMC9485803; doi:10.3389/fphys.2022.911616)

Supplementary Material

1 Validation of the Glucose-Insulin-Glucagon coupled Model

There are different methods of validating ODE based biological models.(1) One method would be to fit data obtained under one set of conditions and validating the model by simulating a second data set obtained for the same individual under different conditions. As data from IIGI experiments for the cohort studied matched to three different OGTT glucose loads was available, validation using this method was possible. The model developed in this paper was validated by simulating the glucose-insulin-glucagon dynamics for IIGI experiments matched to the 25g OGTT glucose profiles of patients with T2D and IIGI experiments matched to 125g OGTT glucose profiles of CS. The simulation was carried out as follows: The glucose infusion, R_{IIGI}, from the 25 g or 125 g equivalent IIGI experiment was used as input and the coupled Equations (1)-(3) were solved using the parameters obtained by fitting the IIGI data matched to the 75 g OGTT as presented in the paper for each patient with T2D and CS respectively.

In the CS simulations, all fit parameters from the 75g equivalent IIGI were kept fixed. The simulated glucose, insulin and glucagon profiles visually match the data from the 125 g IIGI experiment as seen in Supplementary Figure 1.

In the case of patients with T2D, all parameters obtained in the fit of the data from the 75g equivalent IIGI were kept fixed in patients 1 and 7. In the other patients, the glucagon secretion parameter γ_2 and the time delays τ and τ_1 were adjusted manually (Supplementary Table 1). The baseline glucagon levels were significantly different on the two different experimental days (OGTT vs IIGI) for most patients, which could explain why the parameter γ_2 needed to be adjusted to get good fits. The simulated glucose, insulin and glucagon profiles visually match the data from the 25g IIGI experiment as seen in supplementary Figure 2. It is interesting to note that despite discrepancies in baseline glucagon values, good fits were obtained with most of the parameters kept fixed including the glucagon suppression parameter. Only the glucagon secretion parameter had to be adjusted. It is unclear how and why the delays in R_{IIGI} and insulin secretion vary as a function of the glucose load.

1. Hasdemir D, Hoefsloot HCJ, Smilde AK. Validation and selection of ODE based systems biology models: How to arrive at more reliable decisions. BMC Syst Biol. 2015;9(1):1–19.

2 Supplementary Figures and Tables

Supplementary Figure 1. IIGI data corresponding to the 125 g OGTT and simulations using parameters from the fit of the IIGI matched to 75 g OGTT in CS (panels A-F). The visual match between data (black dots) and simulation (blue line) in most individuals is good.

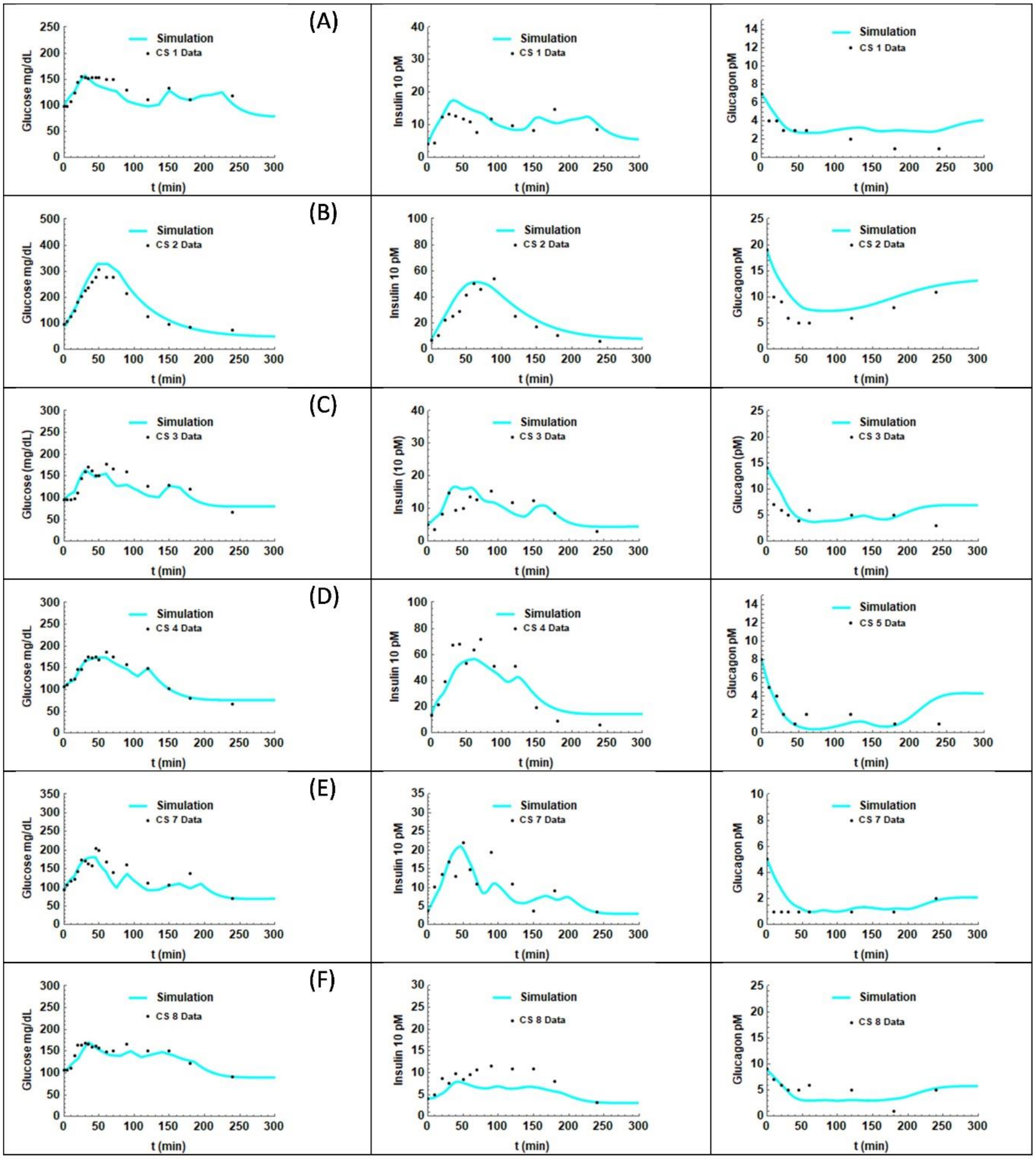

Supplementary Figure 2. Simulation of the IIGI corresponding to the 25 g OGTT using parameters from the fit of the IIGI matched to 75 g OGTT in patients with T2D. The parameters γ_2, τ and τ_1 were manually adjusted for a better fit. The visual match in most patients with T2D is good.

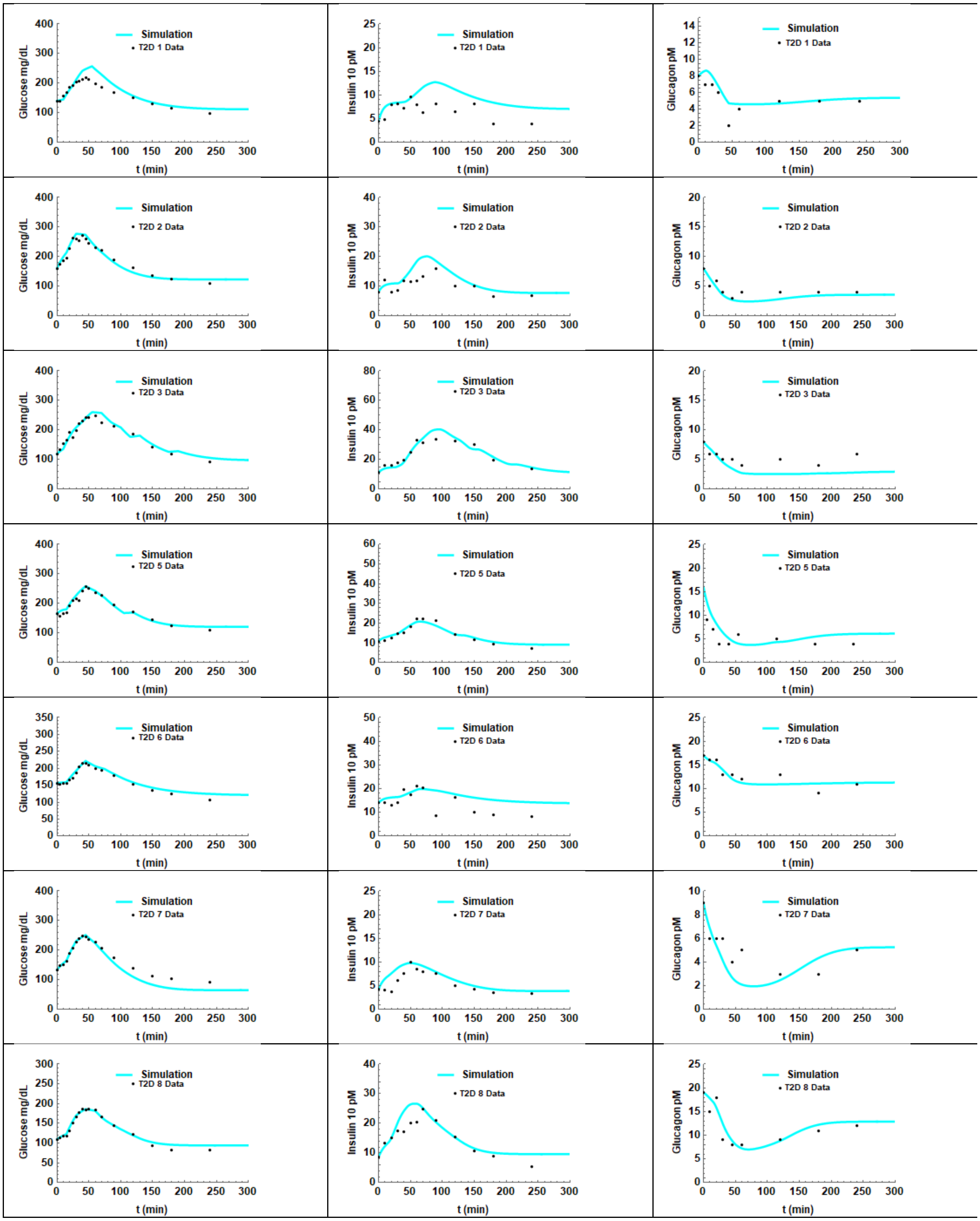

2.1 Supplementary Table 1

Table S1. To obtain a good description of the 25 g OGTT matched IIGI data in T2D patients as seen in Supplementary Figure 2, the following parameters were adjusted: the glucagon secretion parameter, γ_2, and the time delays τ and τ_1. No adjustments were necessary in two patients (1 and 7) where baseline glucose and hormone levels matched on both experimental days. The insulin secretion parameter, γ_1, had to be adjusted for patients 6 and 8.

T2D Patient #	γ_2		τ		τ_1	
	75 g IIGI	25 g IIGI	75 g IIGI	25 g IIGI	75 g IIGI	25 g IIGI
1	2.8	2.8	10	10	30	30
2	3.6	2.2	8	0	30	30
3	1.9	1.2	10	10	35	25
5	4.0	2.1	12	0	30	12
6∗	5.2	3.0	10	0	45	15
7	1.4	1.4	10	10	30	30
8∗	8.6	6.0	0	0	15	0

∗Patient 6: γ_1 5.7−>4.5; Patient 8: $\gamma_1$10.0−>9.0.

Supplementary Figure 3. Plots of the fits obtained with hysteresis Model 2 for patients with T2D in panels A-G. The visual fit of the recovery arm of the insulin secretion profile is not as good as Model 1 particularly in patients 6 and 8 (panels E and G) where the recovery is incomplete. The adjusted R^2 of the overall fits is comparable to model 1.

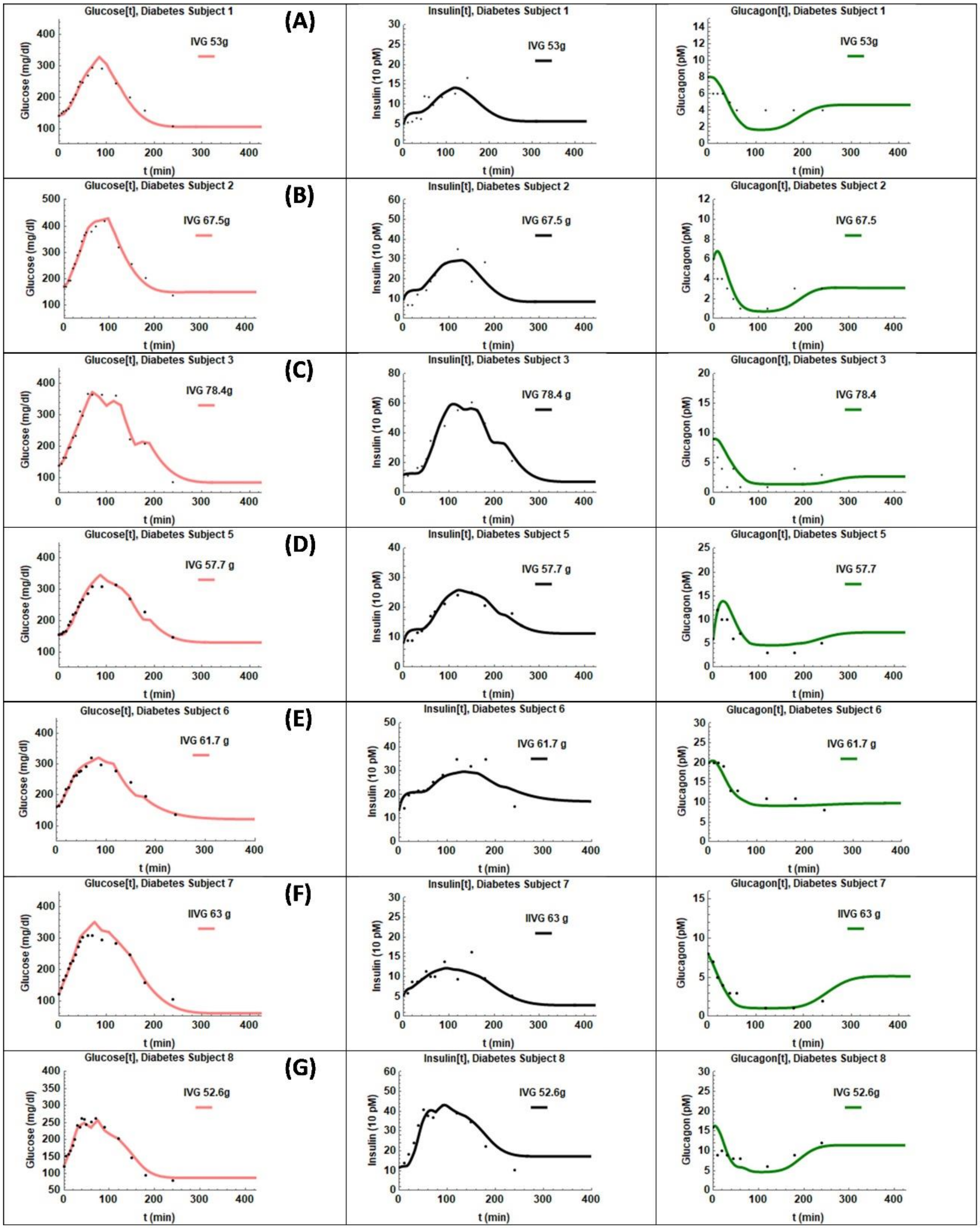

Glucose[t], Diabetes Subject 1
(A)
IVG 53g
Insulin[t], Diabetes Subject 1
Glucagon[t], Diabetes Subject 1
Glucose[t], Diabetes Subject 2
(B)
IVG 67.5g
Insulin[t], Diabetes Subject 2
IVG 67.5 g
Glucagon[t], Diabetes Subject 2
IVG 67.5
Glucose[t], Diabetes Subject 3
(C)
IVG 78.4g
Insulin[t], Diabetes Subject 3
IVG 78.4 g
Glucagon[t], Diabetes Subject 3
IVG 78.4
Glucose[t], Diabetes Subject 5
(D)
IVG 57.7 g
Insulin[t], Diabetes Subject 5
Glucagon[t], Diabetes Subject 5
IVG 57.7
Glucose[t], Diabetes Subject 6
(E)
IVG 61.7 g
Insulin[t], Diabetes Subject 6
Glucagon[t], Diabetes Subject 6
Glucose[t], Diabetes Subject 7
(F)
IIVG 63 g
Insulin[t], Diabetes Subject 7
Glucagon[t], Diabetes Subject 7
Glucose[t], Diabetes Subject 8
(G)
IVG 52.6g
Insulin[t], Diabetes Subject 8
Glucagon[t], Diabetes Subject 8
Glucose (mg/dl)
Insulin (10 pM)
Glucagon (pM)
t (min)

Supplementary Figure 4. Plots of the fits obtained with hysteresis Model 2 for CS subjects (panels A-G). The visual fits of the insulin secretion profiles are similar to that of Model 1. The coefficient of determination (adjusted R^2) of the overall fits is comparable to Model 1.

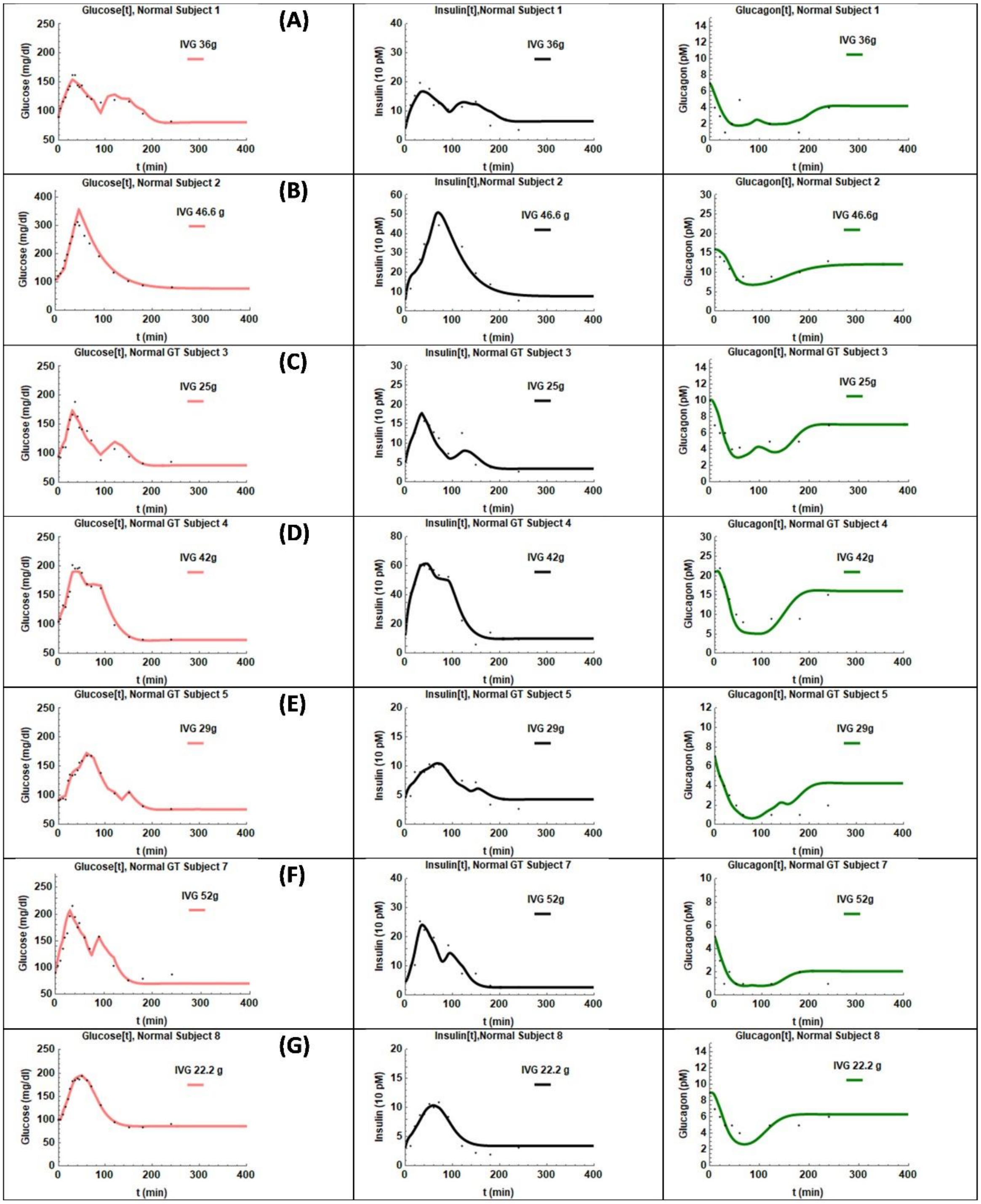

Supplement: Supplementary file 1 [file DataSheet1.pdf]
